# The Lived Experience of Klinefelter Syndrome: A Narrative Review of the Literature

**DOI:** 10.3389/fendo.2019.00825

**Published:** 2019-11-26

**Authors:** Esmée Sinéad Hanna, Tim Cheetham, Kristine Fearon, Cathy Herbrand, Nicky Hudson, Kevin McEleny, Richard Quinton, Eleanor Stevenson, Scott Wilkes

**Affiliations:** ^1^Centre for Reproduction Research, De Montfort University, Leicester, United Kingdom; ^2^Newcastle Hospitals, Newcastle upon Tyne, United Kingdom; ^3^School of Nursing, Duke University, Durham, NC, United States; ^4^School of Medicine, University of Sunderland, Sunderland, United Kingdom

**Keywords:** Klinefelter syndrome, genetic disorders, fertility, patient experience, narrative literature review

## Introduction

Klinefelter syndrome (KS) or 47, XXY is a chromosomal disorder in males. Persons with KS have an additional X chromosome creating karyotype 47, XXY and 46, XY/47, XXY mosaics. According to existing epidemiological studies KS is one of the most common genetic disorders, affecting ~1 in 500 men [see (1, 2)]. Whilst there can be phenotypic variation between individuals, physical traits associated with the syndrome can include small testes, a less muscular body, less facial and body hair, broader hips, and increased breast tissue (1). This physiological background and associated traits can generate questions relating to gender identity and a proportion of KS individuals will not identify as male, instead identifying as female, non-binary or intersex^1^ [see (3)].

Learning difficulties, low self-confidence and issues relating to social interaction are also reported in relation to those with KS (4–6). Whilst a number of physical and developmental issues are therefore associated with KS, infertility is a common feature of the disorder (7). Estimates suggest that over 95% of those with KS are infertile (8), although some men with KS can seek to have biological children using advanced assisted reproductive technologies such as surgical sperm retrieval followed by intracytoplasmic sperm injection (ICSI) (9). Such approaches are however high risk and uncertain, and those with KS may also be faced with decisions about the use of donor sperm, adoption, or remaining childless (10). This review examines the existing psychosocial evidence around the impact of KS, exploring what we know about KS and its relevance for health care for this group.

## Method

In order to identify literature for this review we searched the following key databases: Academic Search Premier, CINAHL, MEDLINE, PsychINFO. The search terms “Klinefelter's syndrome+ Psychosocial^2^” were used to reflect our interest in the psychological and social aspects pertaining to the disorder and specifically to the lived experience of those with KS. The results of these databases were limited to English articles in scholarly academic journals in the last 20 years. There were 47 results generated from this search and identified articles were screened using the inclusion of criteria of being about the patient or lived experience of KS. After screening, 15 results were included for the review, although a further 2 were initially discounted due to not being accessible, but on accessing did not fully meet the inclusion criteria after screening so were not included. Given the small number of results obtained, a Google Scholar search was also conducted, using the same search terms and the first 5 pages of these results were screened (beyond page five revealed the papers were not relevant to the search) which resulted in a further five inclusions. Four further papers were included following identification by reference chaining (11). In total 22 papers were included, as detailed in Table 1 below. These were all papers which met the inclusion criteria specified above and were therefore extracted for the review. An inductive coding approach was adopted as part of the use of qualitative content analysis. This approach is advocated as a useful method when the body of evidence is perceived as limited at the outset of the analysis and when dealing with topics which could be described as sensitive [see (31)]. This inductive approach involves open coding, specifically writing notes and headings during the initial reading phase of the review articles, and these open codes then these headings are grouped into broader “umbrella” categories. As Elo and Kyngas (31) note, “The purpose of creating categories is to provide a means of describing the phenomenon, to increase understanding and to generate knowledge” (:111). From this analysis our overall categories, which we will refer to hear as themes, were then generated, these include; Diagnosis- Issues and timings; Outcomes for those with Klinefelter syndrome^3^; Experiences with health care professionals.

**Table 1:** Study characteristics of included papers.

**References**	**Method, sample size, and country of research**
Abramsky et al. (12)	Phone interviews with health care professionals (*n* = 29) and Questionnaires with parents (*n* = 23) Conducted in the UK
Bhartia and Ramachandran (13)	Patient experience (*n* = 1 auto-ethnographical reflections) and clinician testimonies (*n* = 2). Conducted in the UK
Bojesen and Gravholt (4)	Epidemiological study of KS patients from the UK and Denmark. Cohort of 4,800 patients in the UK and 900 patients in Denmark.
Bourke et al. (14)	Qualitative semi-structured interviews conducted with parents of children with KS (*n* = 15). Conducted in Australia.
Bourke et al. (15)	Practice commentary piece- drawing on practitioner experience in Australia and review of relevant literature.
Close et al. (16)	Triangulated mixed methods study, using semi- structured interviews and online questionnaires with parents of children with KS. Purposive sample of *n* = 40. Conducted in America.
Close et al. (5)	Cross sectional study of boys with KS, samples was *n* = 43. Study included physical examination, hormone analysis and psychosocial questionnaire. Conducted in America.
de Ronde et al. (17)	Questionnaires sent to attendees at Dutch outpatient clinic (*n* = 40)
Geschwind et al. (18)	Discussion of existing studies around neurobehavioral and psychosocial issues and includes pilot data from their study of *n* = 15 adults with KS. Participants completed measures of personality and motivation and measures of problem behaviors. Conducted in America.
Gies et al. (19)	Questionnaire study with clinicians (*n* = 49) and parents (*n* = 18) about fertility preservation. Conducted in Belgium.
Grace (10)	Patient testimony of their experience of diagnosis of KS. Patient based in America.
Groth et al. (20)	Evidence synthesis of studies on KS in PubMED. No details of the number of papers included were provided. Study conducted in Denmark.
Fjermestad and Stokke (21)	Self report data from men (*n* = 53) with sex chromosome aneuploidies (SCA) Data collected via Health Survey–Short Form (SF-36),the Pittsburgh Sleep Quality Index and the Personal Wellbeing Index
Herlihy et al. (22)	Discussion paper based on existing evidence around KS. Conducted in Australia.
Herlihy et al. (23)	Self-completion question with men with KS (*n* = 87) in Australia.
Herlihy et al. (24)	Discussion paper based on review of current evidence around screening for KS. Conducted in Australia.
Nahata et al. (25)	Retrospective study of those diagnosed with KS at Boston Children's hospital. Study conducted in America.
Paduch et al. (26)	Review of existing evidence around KS for urology practice. Study conducted in America.
Skakkebæk et al. (27)	*N* = 132 men with KS were assessed via surveys for demographics, socioeconomic status, health problems and behaviors, sexual function, medical follow-up, and mental and physical quality of life (MQoL and PQoL, respectively). The population group was assessed against a control group (*n* = 313). The study was conducted in Denmark.
Turriff et al. (28)	Self-report survey with people with KS aged 14–75, recruited via online networks (*n* = 310).
Turriff et al. (29)	Online questionnaire with open ended questions, part of a wider study into KS. *N* = 310 completed the study but the responses for the open-ended questions ranged from *n* = 169—*n* = 210 due to incomplete data. Participants were aged 14–75. Conducted in America.
Whitmarsh et al. (30)	Interview study with families of those with genetic disorders. For the KS group they interviewed, six mothers, three fathers, one grandmother (*n* = 10). Study was conducted in America.

## Diagnosis—Issues and Timings

Much of the literature examined discusses the challenges of getting and managing a diagnosis for KS. Fewer than 10% of cases of KS are diagnosed before puberty (4, 25), with only 6% diagnosed before aged 10 and 21% diagnosed before aged 20 (16). The mean age of diagnosis is suggested to be 27 (32) and aspects such as poor learning at school, subsequent challenges around employment and low socio-economic status are believed to be correlated to late or under diagnosis (29). A delay in diagnosis also remains problematic for health aspects including infertility (20). Many boys with KS report growing up with an unexplained sense of “feeling different” (16) and receiving a KS diagnosis, it has been reported as being a “relief” (29). Diagnosis can be a point of acceptance and understanding for patients (10).

Whilst diagnosis can then be a relief for those with KS, literature relating to the experience of parents of boys with KS shows that diagnosis can be uncertain and complex which can be a source of frustration for parents (14, 30). Even though parents may struggle to obtain a diagnosis for their children, particularly where there is an absence of “typical” physical symptoms associated with KS, they are not always well prepared to receive a genetic diagnosis when it is ultimately obtained (13, 14).

## Outcomes for Those With Klinefelter Syndrome

Quality of life (QoL) outcomes are reported as being worse for men with KS than for the general population (17, 22, 23, 25, 27, 28). There are also higher rates of anxiety and depression found in people with KS (18, 25) and sleep related problems (21). The phenotypic severity influences the psychosocial outcomes for patients (27) and a higher number of physical features attributed to KS inversely relates to QoL (5).

Turriff et al. (29) found that infertility along with psychosocial challenges were viewed as a major issue for those with KS. It is suggested that 50% of adult men with KS will yield viable sperm as a result of advances in reproductive technologies (26). There is however a desire from paediatricians and parents of KS children to see fertility preservation being used for minors who have KS (19). Parents are often concerned about sexuality, masculinity and fertility after a diagnosis, with the fathers of KS boys seen as particularly concerned about their son's sexual development and functioning (14). Evidence suggests that gender identity can be an issue for those with KS, with some reporting they neither feel, or look either masculine or feminine (23).

Physical health outcomes for those with KS can include lower physical activity levels and higher BMIs (27) as well as an increased risk of osteoporosis, diabetes as well as breast and other cancers (4). This increases both morbidity but also premature mortality (27) and those with KS have a decreased life expectancy of between 2–6 years (15). Whilst there is no cure for KS, many of these health issues are viewed as being best managed through early diagnosis of KS and relevant ongoing healthcare (15, 20).

## Experiences With Health Care Professionals (HCPs)

There is seen to be widespread lack of knowledge about KS by HCPs (5, 14, 29), with a “haphazard” approach taken to the informing of parents around the diagnosis of KS (12). Information given to those who have KS is seen to be inconsistent and HCPs are often viewed as lacking insight into the realities of KS (29). Given that KS is not heritable, parents may lack knowledge of what KS is, demonstrating the need for good quality professional support to plan for the care of their children with KS (14, 16). However, common misconceptions around KS are reported as being conveyed from HCPs, such as parents being told their sons are more likely to be gay as a result of having KS (14) despite the contested nature of evidence about differential rates of people identifying as gay among those with KS when compared to the general population (23, 27).

Knowledge amongst healthcare professionals around treatment options is also now seen to be outdated (14) and not evidence based, due to lack of research around testosterone replacement or other management interventions (4, 5). The existing literature suggests that those with KS would be best served by multidisciplinary and coordinated health care (20, 27, 29, 32) supported by more training and education for HCPs (14). In light of a lack of quality information forthcoming from HCPs, parents of children with KS are seen to turn to the internet for help and advice (5), and others have noted the importance of support groups for those with KS, as a mechanism to help with the uncertainty of what having KS will mean for their lives (15).

## Discussion

This narrative review suggests that a lack of or late diagnosis remains a critical problem in relation to KS. Whilst prenatal screening techniques may improve future diagnosis (33), current low levels of diagnosis remain problematic, particularly for the possibility of improving physical and mental health outcomes (25). This is particularly important as those with KS are reported to have poorer health outcomes than the general population across a range of measures, including quality of life (23, 25, 27) and comorbidities result in a decreased life expectancy for those with the disorder. The perception that all persons with KS will demonstrate “textbook” signs is viewed as compromising the ability of patients to obtain a diagnosis (34). Early diagnosis allows for more extensive options for children and adolescents to preserve their fertility, which is seen as one of the key concerns for patients, although this remains an area in need of further research (19). Diagnosis itself can be a relief for patients, which is similar to other long-term health conditions [see (35–37)] although the literature details that uncertainty can also spring from a KS diagnosis, perhaps connected to the perceived lack of knowledge by HCPs reported within the literature.

The experience with healthcare for persons with KS is described as poor (5, 14, 29), ranging from a lack of information to misinformation, due to a perceived lack of expertise among HCPs around KS. There is a consensus in the literature around the importance and value of the multidisciplinary team as a means of providing care to KS patients (20). Coordinated approaches to care are currently seen to be lacking despite evidence of the effectiveness of such approaches being noted in relation to other illnesses (38, 39). Questions of gender identity are noted within the literature (23) but not extensively explored; how those with KS identify and how this then intersects with their experiences of healthcare remains an important area for future consideration.

Given the prevalence of KS within the population, greater research focus on the disorder in the future, particularly in relation to reproductive health and the psychosocial impact of KS, would have a significant impact for patients and their families. There are inevitably limitations to a short review of this nature, and not all papers which may be relevant to KS, particularly those which are more clinically focused [such as (32)] appeared within our search, thereby illustrating a well-recognized limitation of literature keyword search based review algorithms. The voices of those with KS appear to be currently lacking from the literature, which could be further marginalizing, so future research should attempt to capture the lived experience of those with KS and use participatory methods where possible to embed this lived experience centrally within research. Developing a priority setting partnership for those with KS to identify and rank key research areas for the future would be fruitful, and co-production of research agendas would help with inclusion of this otherwise hidden group. Attempts to move forward research and care for those with KS should then begin with a central focus on what matters to those with KS and seek to make positive improvements to their diagnosis, outcomes and encounters with healthcare professionals.

## Author Contributions

EH led the analysis and drafting and all other authors equally contributed to the writing, editing, and refining of the work. All authors contributed to the preparation of the manuscript.

### Conflict of Interest

The authors declare that the research was conducted in the absence of any commercial or financial relationships that could be construed as a potential conflict of interest.

## Footnotes

^1^Whilst we refer to men within this paper, given that primarily those with KS will identify as male, we are aware that not all will identify as male, and have chosen this terminology for clarity and to reflect the medical literature on this topic.

^2^We have chosen to use Klinefelter syndrome as a descriptor of the disorder within our work, in line with NHS guidance (https://www.nhs.uk/conditions/klinefelters-syndrome/), but it is also widely called Klinefelter's syndrome. The papers we have included use of a mix of “Klinefelter” and “Klinefelter's”. For our search using “Klinefelter syndrome+ psychosocial” brought up only 11 results across all time in the databases we searched so despite our preference to call it Klinefelter syndrome we have chosen to use the search terms “Klinefelter's syndrome + psychosocial” in order to maximise results as they then include “Klinefelter” and “Klinefelter's.”

^3^The concept of outcomes from the analysis within this paper, and these relate to psychosocial outcomes within this paper, as per the aims and objectives of the article, but we do for ease of discussion refer to this as simply “outcomes” within the paper.
